# Seasonal changes in endoplasmic reticulum stress and ovarian steroidogenesis in the muskrats (*Ondatra zibethicus*)

**DOI:** 10.3389/fendo.2023.1123699

**Published:** 2023-02-07

**Authors:** Wenjing Lu, Qingjing Gao, Jinlan Wei, Wenqian Xie, Haolin Zhang, Zhengrong Yuan, Yingying Han, Qiang Weng

**Affiliations:** ^1^ College of Biological Science and Technology, Beijing Forestry University, Beijing, China; ^2^ School of Public Health, Tongji Medical College, Huazhong University of Science and Technology, Wuhan, China

**Keywords:** endoplasmic reticulum stress, muskrat, ovary, steroidogenesis, unfolded protein response

## Abstract

Many studies have shown roles for endoplasmic reticulum stress (ERS)/unfolded protein response (UPR) signaling cascades with ovarian folliculogenesis, and oocyte maturation. In this study, we investigated seasonal changes in ERS and ovarian steroidogenesis in the muskrats (*Ondatra zibethicus*) during the breeding season (BS) and non-breeding season (NBS). There were noticeable seasonal variations in the weight and size of muskrat ovaries with values higher in the BS than that in NBS. The circulating luteinizing hormone (LH), follicle-stimulating hormone (FSH), 17β-estradiol, and progesterone of the female muskrats were higher during the BS. The RNA-seq data of ovaries during different seasons revealed 2580 differentially expressed genes, further analysis showed a prominent enrichment of ERS-related pathways and ovarian steroidogenesis pathway. Immunohistochemical results showed that GRP78 and steroidogenic enzymes (P450scc, 3β-HSD, P450c17, and P450arom) existed in the various kinds of cells in muskrat ovaries during the BS and NBS. In ovaries from the BS, the mRNA levels of P450scc, P450arom, P450c17, and 3β-HSD were considerably higher. Furthermore, the expression levels of oxidative stress-related genes (SOD2, CAT, and GPX1) and UPR signal genes (Bip/GRP78, ATF4, ATF6, and XBP1s) were increased strikingly higher during the BS in comparison with the NBS. However, the mRNA levels of CCAAT-enhancer-binding protein homologous protein (CHOP) and caspase-3 had no considerable difference between the BS and NBS. Taken together, these results suggested that UPR signaling associated with the seasonal changes in ovarian steroidogenesis is activated in the BS and the delicate balance in redox regulation is important for seasonal reproduction in the muskrats.

## Introduction

The endoplasmic reticulum (ER) is the primary cell organ accountable for various particular cellular processes such as the synthesis and folding of secretory or membrane proteins, fatty acid and steroid biosynthesis, and Ca^2+^ storage (1, 2). Endoplasmic reticulum stress (ERS) is induced when there is an imbalance between the volume in the ER and the protein folding load, it causes a buildup of unfolded or improperly folded proteins (3). Numerous physiological and pathological processes, including excessive secretion requirement, damaged calcium homeostasis, changed lipid homeostasis, oxidative stress, and production of the mutant protein linked to disease all contribute to the induction of ERS (4–7). When ERS occurs, the three embranchments of the unfolded protein response (UPR) signaling are decoupled from glucose-regulated protein 78 (GRP78) and activated, which are protein kinase RNA (PKR)-like ER kinase (PERK), inositolrequiring enzyme 1 (IRE1), and activating transcription factor 6 (ATF6) (8, 9). PERK phosphorylates eukaryotic initiation factor 2α (eIF-2α), which inhibits the entry of new proteins into the endoplasmic reticulum and attenuates translational initiation once activated. Phosphorylated eIF-2α activates activating transcription factor 4 (ATF4) transcriptional translations, which enhances antioxidant response and improves the folding capacity of the ER (10). Protein synthesis is inhibited by the phosphorylation of IRE1, and splicing X-box-binding protein 1 (Xbp1s) transcription factors are activated for chaperone activities (11). In theory, UPR restores ER homeostasis firstly as soon as possible by promoting protein degradation and transport, modification of misfolded proteins, and inhibition of protein synthesis, allowing the cell to resume normal physiological activities (12). However, an excessive ERS induces activation of CCAAT-enhancer binding protein homolog (CHOP), Jun N-terminal kinase (JNK), and cleaved caspase 3, resulting in cell death (13). Recent studies have revealed that the ERS response has a crucial influence on the regulation of female mammalian reproductive processes such as follicular development, follicular atresia, embryo attachment, and steroidogenesis (14, 15).

The ovaries provide two functions in the reproductive system of animals. One of them is the reproductive function, which is in charge of maturing and releasing oocytes for fertilization, and the other is the endocrine function, which is in charge of producing and secreting sex hormones including progesterone and estrogen (16). The steroid hormone synthesis and secretion is a complex process that is closely regulated and controlled by follicle-stimulating hormone (FSH), luteinizing hormone (LH), cytokines, and some protein enzymes (17). To begin, steroidogenic acute regulatory (StAR) proteins must be used to transport cholesterol to the inner mitochondrial membrane from the outer mitochondrial membrane (18). Then, pregnenolone is produced under the action of the cytochrome P450 side-chain cleavage enzyme (P450scc) from cholesterol, which starts that steroidogenesis process. The 3β-hydroxysteroid dehydrogenase enzyme (3β-HSD) converts pregnenolone to progesterone when it exits the mitochondria and enters the ER. Progesterone is released from granulose cells and transferred into thecal cells; there it is converted to testosterone under the action of the cytochrome P450 17-hydroxylase/17,20-lyase (P450c17) as granulose cells cannot produce androgen. After the testosterone has been moved back to granulose cells, the aromatase cytochrome P450 (P450arom) catalyzes them to produce estrogens in ER (19). The critical enzymes in the hormone synthesis pathway include P450scc, 3β-HSD, P450c17, and P450arom, and their mutations will cause a shortage of steroid hormones. The follicular and luteal cells are rich in ER to maintain cholesterol synthesis, and the normal function of ER provides the sufficient substrate for the synthesis of estrogen and progesterone (20). Therefore, we speculate that the UPR signaling, activated by ERS conditions, may be involved in the regulation of steroid hormone synthesis.

Muskrat (*Ondatra zibethicus*) is a semi-aquatic, medium-sized herbivore, which is native to North America (21). The muskrat breeds seasonally, with its sexual activity occurring from March to October (22). From March, when the breeding season begins, the scent glands of male muskrats begin to develop and secrete the musky substance, whose function is mainly to transmit excitement information through scent to induce the female muskrats to estrus (23). During the breeding season, males are in constant estrus while females are in cyclic estrus (24). The estrus cycle of female muskrats is generally 15-22 days, including 1-2 days in proestrus, 2-4 days in the duration of estrus, 1-2 days in postestrus, and 13-20 days in diestrus, and females after March have mature follicles and most stop estrus by early October (25). The gestation period of muskrats is very short, only 27 to 28 days, and each litter can give birth to 6 to 9 litters (24). A female can theoretically reproduce 20 to 25 muskrats per year. This reproductive characteristic makes the muskrat a good model for studying seasonal changes in gonadal functions. Numerous studies on muskrats have shown that the levels of steroid hormones are significantly elevated during the breeding season, accompanied by elevated levels of steroidogenic enzymes (26–28). For the past few years, a growing number of studies have shown an inextricable link between UPR signal and female reproduction (29). However, the changes in ERS-mediated UPR signals during different breeding seasons in seasonally breeding mammals are unclear. Accordingly, we investigate the expression patterns of UPR signal genes and steroidogenic enzymes in muskrat ovaries and the concentrations of steroid hormones of the female muskrats in this study, to gain in-depth knowledge of the relationship of UPR signal regarding seasonal variations in the ovarian steroidogenesis of female muskrats.

## Materials and methods

### Animals and tissues collection

18 adult female muskrats aged 12 months to 24 months were purchased from the Xinji Muskrats Breeding Farm in Hebei Province, China in May (breeding season, BS, n = 9) and December (non-breeding season, NBS, n = 9). The Guidelines for the Care and Use of Laboratory Animals in China were followed for all research. The Ethics Committee of Experimental Animals at Beijing Forestry University also gave its approval to this work. Animals were anesthetized with diethyl ether and blood samples were rapidly collected from the carotid artery as previously described (26). Ovaries were quickly removed and dissected from the female muskrats. We immediately measured the weight, length, and width of each ovary after excision. One side of the tissues was immediately immersion-fixed in 0.05 M phosphate-buffered saline (PBS, pH 7.4) containing 4% paraformaldehyde and preserved in 70% ethanol for histological and immunohistochemical analyses as previously described (30), and the other side was swiftly frozen in liquid nitrogen and kept at -80°C.

### Histology

The ovarian samples underwent ethanol series dehydration before being paraffin-embedded. Serial (5 μm) sections were put on slides that were poly-L-lysine coated. Hematoxylin-eosin staining was used on several sections (HE). The dyed slides were examined histologically under a microscope. Per Section, six arbitrary vision areas were chosen to observe. The remaining portions underwent immunohistochemistry processing.

### Transcriptome analysis

Total RNA was extracted from muskrat ovaries by means of the TRIzol reagent (CWBIO, China). The quantity and concentration of RNA were assessed using the NanoDrop 8000 (Thermo, USA). The directions are strictly followed during every procedure. RNA quality was evaluated using a 2100 Bioanalyzer (Agilent Technologies, USA), and RNA integrity number (RIN) ≥ 6.5 was found for three RNA samples per group. High-throughput sequencing was conducted on the Illumina novaseq 6000 platform in accordance with the instructions once library creation and inspection were complete.

The quality of the raw reads from the muskrat ovarian samples was evaluated using the FastQC software. Calculated from the raw data of each sample were the read count, uncertain bases, Q30, and GC content percentage. Clean readings were produced by eliminating adapter- or poly-N-containing reads as well as reads with more than 50% of low-quality (Q-value ≤ 10) bases. The DESeq2 tool in R (v4.1.2) was used to classify the differentially expressed genes (DEGs) (31). The term “differentially expressed” was applied to genes with adjusted *p-value* ≤ 0.05 and |Log_2_FoldChange| ≥ 1. According to the GO database, functional annotation and enrichment analysis of DEGs were performed by the GOseq software to classify the primary biological functions (32). The pathways of DEGs were analyzed by using the KOBAS software (33), and the primary functions of these genes in muskrat ovaries were recognized.

### qRT-PCR

According to the above method of RNA extraction and the extracted RNA is reversely transcribed into cDNA. Then, GRP78, ATF4, ATF6, XBP1s, CHOP, CASP3, SOD2, CAT, GPX1, P450scc, 3β-HSD, P450c17, and P450arom were analyzed by qRT-PCR to measure its expression. The primer sequences for mRNA are summed up in **Table 1**. To standardize the transcription levels of each gene, β-actin was used as an endogenous control.

**Table 1:** Primers sequence used for mRNA qRT-PCR.

Gene name	Forward Primer	Reverse Primer
GRP78	5’-GTGCCCACCAAGAAGTCTCA-3’	5’-ATTTCTTCAGGGGTCAGGCG-3’
ATF4	5’-GACACCGGCAAGGAGGATG-3’	5’-TGGCCAATTGGGTTCACTGT-3’
ATF6	5’-ATCACCTGCTATTACCAGCTACCAC-3’	5’-TGACCTGACAGTCAATCTGCATC-3’
XBP1s	5’-GTCCGCAGCACTCAGACTAC-3’	5’-AGGGAGGCTGGTAAGGAACT-3’
CHOP	5’-GTCACAAGCACCTCCCAAAGCC-3’	5’-CGCACTGACCACTCTGTTTCCG-3’
CASP3	5’-GAAGATACCAGTGGAGGCCG-3’	5’-CGCGTACAGTTTCAGCATGG-3’
SOD2	5’-CGGGGGCCATATCAATCACA-3’	5’-GGTCCTGATTAGAGCAGGCG-3’
CAT	5’-ATGGCTATGGCTCACACACC-3’	5’-TGAGGCCAAACCTTGGTCAG-3’
GPX1	5’-CATCATTTGGTCCCCGGTGT-3’	5’-TTGCTAGGCTGCTTGGACAG-3’
P450scc	5’-CAGATGCCTGGAGGAAAGAC-3’	5’-GATGGACTCAAAGGCAAAGC-3’
3β-HSD	5’-GTCATGATACTTGCGGCCCT-3’	5’-CCATTCCTTGCTCAGGGTGC-3’
P450c17	5’-GCCACTATCCGAGAAGTGCT-3’	5’-GCAAGTAACTCTGCGTGGGT-3’
P450arom	5’-ACACCATGTCCGTCACTCTG-3’	5’-GCCGTCAATCACGTCATCCT-3’
β-Actin	5’-GACTCGTCGTACTCCTGCTT-3’	5’-AAGACCTCTATGCCAACACC-3’

### Immunohistochemistry

After the dewaxing process, muskrat ovaries were slowly washed 3 times with phosphate buffer (0.01M) for 5 minutes each. Samples were added to citrate buffer (10 mM) and heated to repair the antigen, naturally cooled to room temperature then washed as in the previous step. Block with 10% normal goat serum prior to incubation with primary antibody. Incubate the primary polyclonal antibodies against GRP78 (ab21685, Abcam, Cambridgeshire, United Kingdom), P450scc (bs-10099R), 3β-HSD (bs-3906R), P450c17 (bs-3853R), and P450arom (bs-0114R) (Bioss Biotechnology, Beijing, China) at 4°C for 12 hours. After washing off the primary antibody, the tissues were successively incubated with biotin-labeled secondary antibody and horseradish peroxidase working solution (SP-0022, Bioss Biotechnology, Beijing, China) for 30 minutes, respectively, and then visualized with 3, 3-diaminobenzidine (Wako, Tokyo, Japan) solution at room temperature and terminated with distilled water. Finally, the nuclei were re-stained with hematoxylin and tissues were dehydrated and sealed with neutral balsam.

### Hormone assay

The female blood samples were centrifuged at 3000×g for 15 minutes at 4°C which from both seasons. The enzyme-linked immunosorbent assay (ELISA) kits were used to analyze supernatant right away to measure the levels of the plasma hormones (FSH ELISA Kit, CSB-E06869 r; LH ELISA Kit, CSB-E12654 r; 17β-estradiol ELISA Kit, CSB-E05110 r; progesterone ELISA Kit, CSB-E07282 r, Cus-bio Biotech Co., Ltd., Wuhan, China). All ELISA kits have a CV (coefficient of variation) that is under 15% both within and between experiments.

### Statistical analysis

At least three times each of the experiments were repeated. R software was used to conduct statistical analysis. After determining the homogeneity of variance with Levene’s test using the SPSS 26.0, data were analyzed using the Student’s t-test. The data are served as the means ± standard errors (SE), and a *P* < 0.05 was regarded as statistically considerable.

## Results

### Morphological observation and histological features

**Figure 1:**
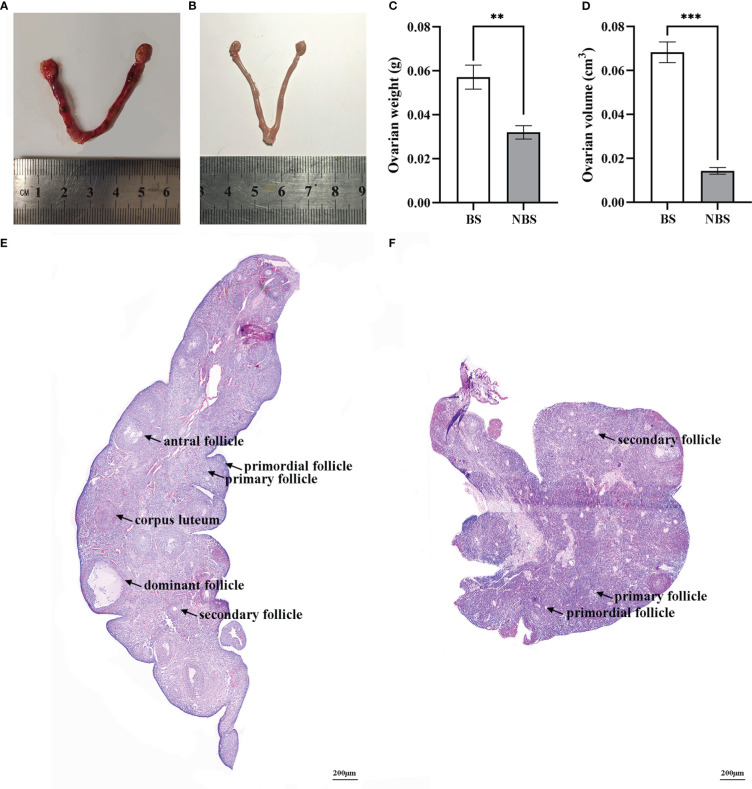
Morphological and histological features of ovarian tissues of the muskrats during the breeding and non-breeding seasons. The morphology of the ovaries and uterus of the muskrats during the breeding season **(A)** and non-breeding season **(B)**; Marked seasonal differences were observed in ovarian weight **(C)** and volume **(D)**. HE staining was performed for the ovaries of the breeding season **(E)** and non-breeding season **(F)**. BS, breeding season; NBS, non-breeding season. Scale bars represent 200 μm **(E, F)**. *Statistically significant values (**P < 0.01, ***P < 0.001).

**Figure 1** displayed the morphological and histological observations of the ovarian tissues of female muskrats both from the BS and the NBS. We observed a remarkable reduction in the morphological size of the ovaries and the uterus of the muskrats from the NBS (**Figure 1B**) in comparison with the BS (**Figure 1A**). Compared to the ovaries from the BS, the average weight and volume of the ovaries from the NBS were strikingly decreased (**Figures 1C, D**). In the muskrat ovaries from the BS, we could observe various types of follicles as well as the corpus luteal (**Figure 1E**), while primary and secondary follicles constituted the majority of the NBS, with only a few tertiary follicles (**Figure 1F**).

### Identifying differentially expressed genes

**Figure 2:**
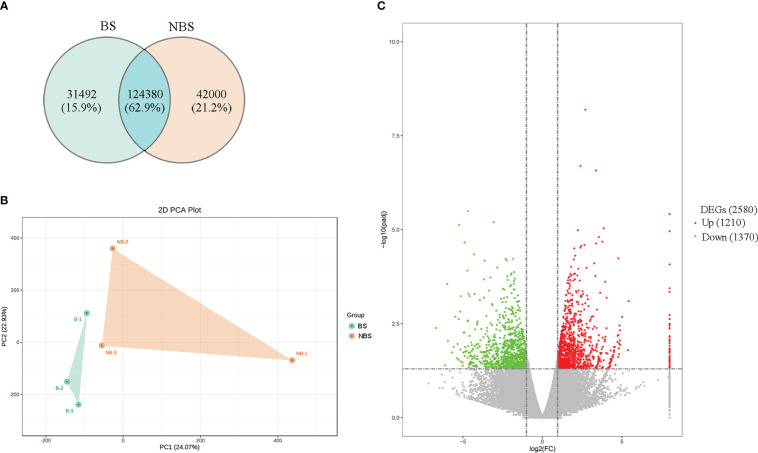
**(A)** The Venn map of expressed genes in the ovaries of breeding and non-breeding seasons. **(B)** PCA score plot of ovary transcriptomes. BS, breeding season; NBS, non-breeding season. Green point, samples from BS; Orange point, samples from NBS. **(C)** Volcano map of differential expressed genes (DEGs) in the ovaries of breeding and non-breeding seasons.

The ovarian tissues of the muskrats from different seasons were identified with 197,872 transcripts in total, of which 155,872 transcripts were found in ovaries from the BS and 166,380 transcripts were found in the NBS, with 124,380 transcripts for both groups (**Figure 2A**). Following that, principal component analysis (PCA) was used to analyze muskrat ovaries. The PCA score plots (**Figure 2B**), which showed a clear distinction between the BS and NBS groups, generally indicated that the samples from the various groups were split into two halves. By converting FPKM to DEGs, 2580 DEGs were identified, allowing researchers to more clearly grasp how the BS and NBS groups differed in their gene expression. The volcano diagram displays the specific results (**Figure 2C**). Compared with ovaries from the NBS, 1210 genes were up-regulated and 1370 genes were down-regulated in ovaries from the BS.

### GO and KEGG pathway analysis

**Figure 3:**
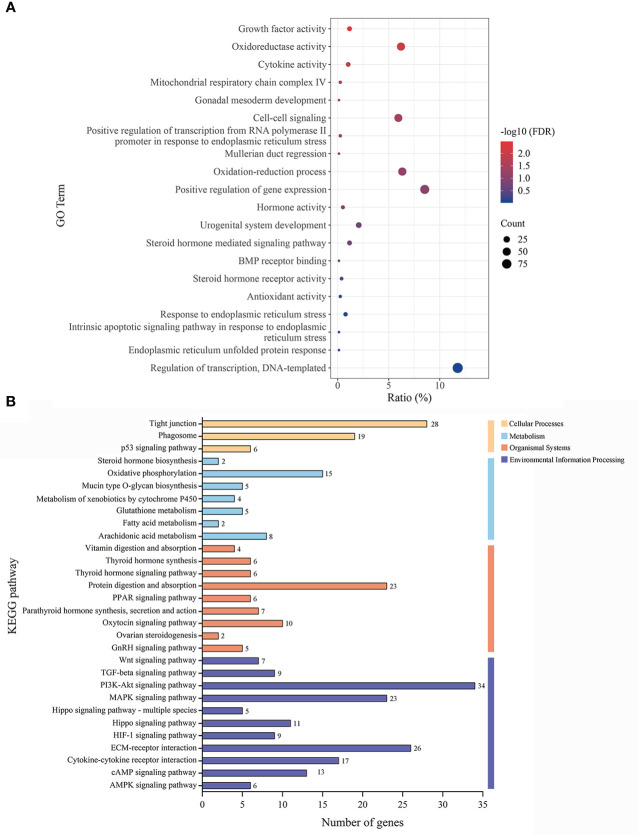
**(A)** The GO classification diagram of the DEGs in ovarian tissues of the muskrats during breeding and non-breeding seasons. **(B)** The distribution of KEGG enrichment pathways in the DEGs of ovarian tissues of the muskrats.

To investigate the probable mechanisms of seasonal reproduction in females, we screened for the 2580 DEGs in the ovaries of the muskrats from the BS and NBS. In particular, there were 38 genes identified related to ER (**Supplementary Figure S1**). As shown in **Figure 3A**, we performed the GO analysis that revealed significant participation of the ERS pathway, including “endoplasmic reticulum unfolded protein response”, etc. The significantly enriched KEGG pathways were listed in **Figure 3B**. Notably, “ovarian steroidogenesis” was identified as the significant pathway in the ovaries. Meanwhile, “PI3K-Akt signaling pathway” and “MAPK signaling pathway” were also significantly enriched.

### Immunolocalizations of Bip/GRP78

**Figure 4:**
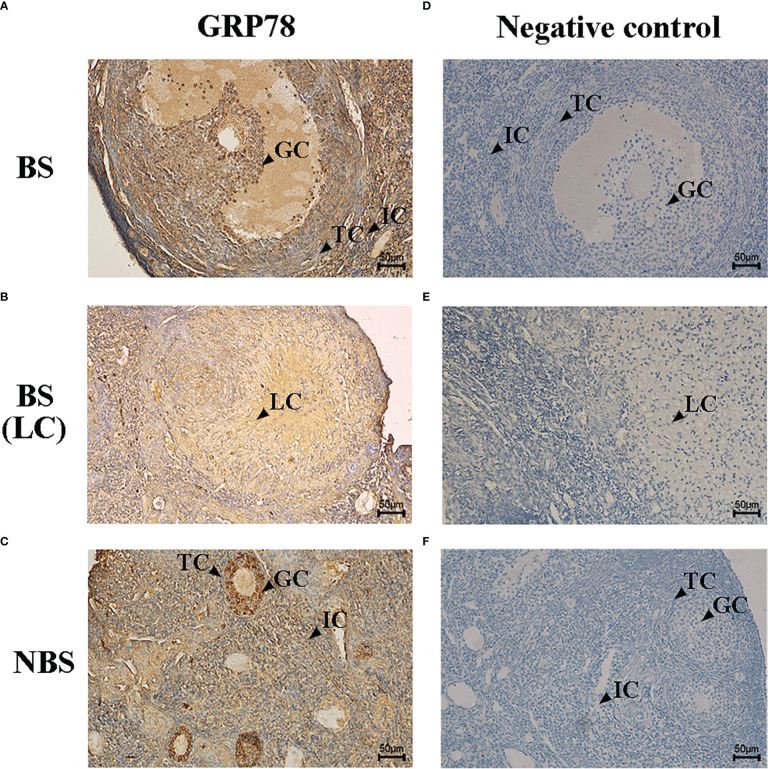
Immunohistochemical localization of GRP78 in ovaries of the muskrats during breeding season **(A, B)** and non-breeding season **(C)**. The negative control of the ovaries sections in breeding season **(D, E)** and non-breeding season **(F)**. BS, breeding season; NBS, non-breeding season. GC, granulosa cells; TC, theca cells; IC, interstitial cells; LC, luteal cells. Scale bars represent 50 μm.

The immunohistochemical localization of GRP78 in the ovaries of the female muskrats were shown in **Figure 4**. In the ovaries from the BS, the immunoreactivity of GRP78 was substantially concentrated in the interstitial cells, but we also observed the immunohistochemical localizations of GRP78 in the mature follicles and luteal cells (**Figures 4A, B**). In the ovaries from the NBS, we noticed that GRP78 staining was positive in granulosa cells (**Figure 4C**). **Table 2** provides a summary and quantification of the staining data that were acquired from the images.

**Table 2:** Relative abundance of Bip/GRP78, ATF4, ATF6, XBP1s, P450scc, 3β-HSD, P450c17, and P450arom in ovaries of the muskrats during the breeding and non-breeding seasons.

Antibodies	Breeding season				Non-breeding season		
	GC	TC	IC	LC	GC	TC	IC
GRP78	+ +	+ +	+ + +	+ +	+ + +	+	+
P450scc	+	+ + +	+ +	+ +	+	+	+
3β-HSD	+ + +	+ + +	+ + +	+ +	+	+	–
P450c17	+	+ + +	+ + +	+ +	+	+ +	+
P450arom	+ +	+	+	+	+	–	–

GC, granulosa cells; TC, theca cells; IC, interstitial cells; LC, luteal cells. –, negative staining; +, positive staining; ++, strong positive staining; +++, very strong positive staining.

### Immunolocalizations of P450scc, 3β-HSD, P450c17, and P450arom

**Figure 5:**
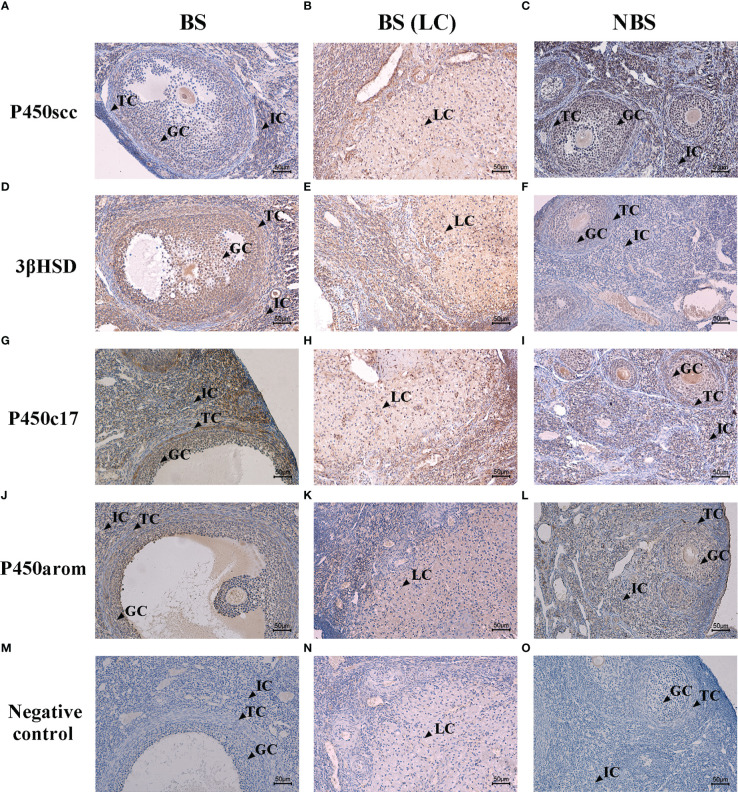
Immunohistochemical localization of P450scc **(A–C)**, 3β-HSD **(D–F)**, P450c17 **(G–I)**, and P450arom **(J–L)** in ovaries of the muskrats during breeding and non-breeding seasons. The negative control of the ovaries sections in breeding season **(M, N)** and non-breeding season **(O)**. BS, breeding season; NBS, non-breeding season. GC, granulosa cells; TC, theca cells; IC, interstitial cells; LC, luteal cells. Scale bars represent 50 μm.

The immunohistochemical localizations of P450scc, P450arom, P450c17, and 3β-HSD in the ovaries of the female muskrats were displayed in **Figure 5**. The immunoreactivity of P450scc and P450c17 was found to be localized mainly in cells in the follicles of ovaries from the BS (**Figures 5A, J**). Comparing the BS to the NBS, the immunohistochemical localizations of 3β-HSD were also found in these cells in the follicles (**Figures 5D–F**). In ovaries from the NBS, the immunostaining intensity is generally reduced. In ovaries from BS, P450arom was found in a variety of cells, with the strongest positive signals in granulosa cells (**Figure 5J**). In ovaries from the NBS, the granulosa cells were where the immunoreactivity of P450arom was primarily located (**Figure 5L**). The negative control panel showed no signal (**Figures 5M–O**). **Table 2** provides a summary and quantification of the staining data that were acquired from the images.

### Seasonal expressions of UPR signal genes and steroidogenic enzymes

**Figure 6:**
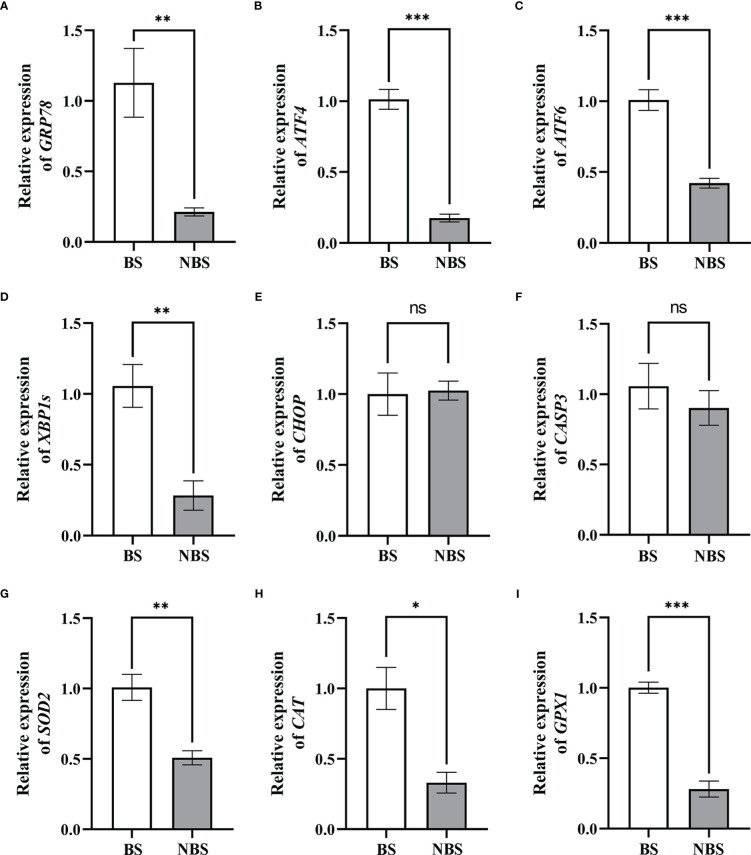
The mRNA seasonal expressions of GRP78 **(A)**, ATF4 **(B)**, ATF6 **(C)**, XBP1s **(D)**, CHOP **(E)**, CASP3 **(F)**, SOD2 **(G)**, CAT **(H)**, and GPX1 **(I)** in the ovaries of the muskrats. BS, the breeding season; NBS, the non-breeding season. The sample number is 3 for each group. The error bars represent means ± SEM. Significance is indicated by *P < 0.05, **P < 0.01, ***P < 0.001, and ns, not significant.

The mRNA levels of UPR signal genes and oxidative stress-related genes were quantified by qRT-PCR, and the results were shown in **Figure 6**. We observed that the expressions of ER stress markers in ovaries from the BS were increased, such as GRP78 and XBP1s, compared with the NBS (*P* < 0.01) (**Figures 6A, D**). Meanwhile, the expressions of UPR signal genes (ATF4 and ATF6) activated by ERS also increased strikingly in ovaries from the BS (*P* < 0.001) (**Figures 6B, C**). Consistently, the mRNA levels of SOD2, CAT, and GPX1 in the ovaries from the BS were markedly higher than those of the NBS (**Figures 6G–I**). However, the transcription levels of CHOP and CASP3 in muskrat ovaries were not obviously different between the BS and the NBS (**Figures 6E, F**).

**Figure 7:**
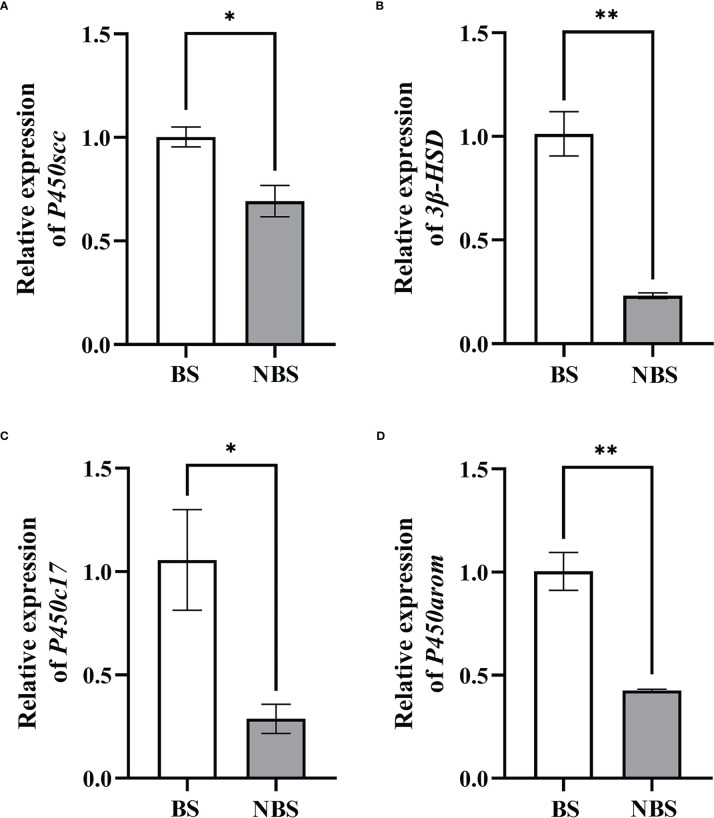
The mRNA seasonal expressions of P450scc **(A)**, 3β-HSD **(B)**, P450c17 **(C)**, and P450arom **(D)** in the ovaries of the muskrats. BS, breeding season; NBS, non-breeding season. The sample number is 3 for each group. The error bars represent means ± SEM. *Statistically significant values (*P < 0.05, **P < 0.01).

In the same way, the transcription levels of P450scc, P450arom, P450c17, and 3β-HSD were detected by qRT-PCR, and the results were shown in **Figure 7**. Compared to the ovaries from the BS, the levels of P450scc and P450c17 of the female muskrats were all remarkably lower than those from the NBS (*P* < 0.05) (**Figures 7A, C**). In the same way, the transcription levels of 3β-HSD and P450arom in muskrat ovaries reduced strikingly in ovaries from the BS to the NBS (*P* < 0.01) (**Figures 7B, D**).

### Seasonal changes in FSH, LH, progesterone, and 17β-estradiol concentrations

**Figure 8:**
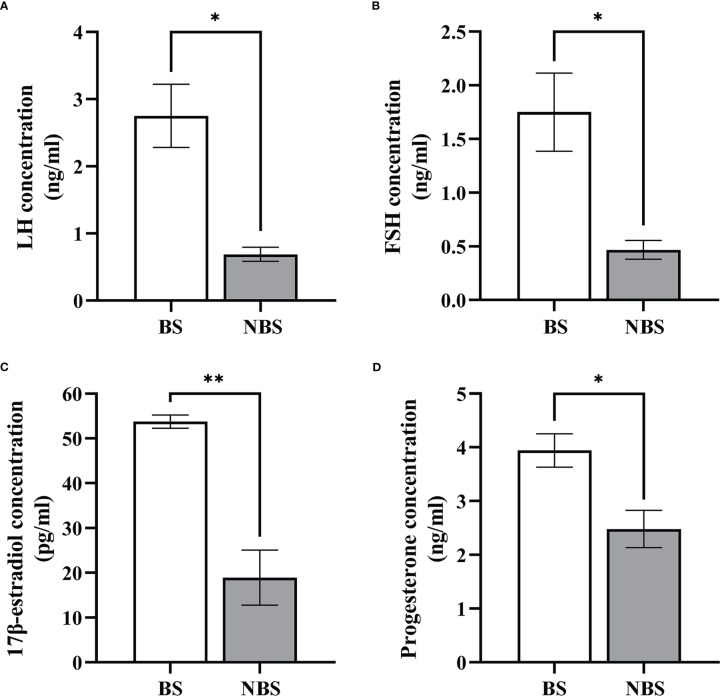
Seasonal change of the concentration of LH, FSH, estradiol-17β, and progesterone. Serum LH **(A)**, FSH **(B)**, 17β-estradiol **(C)**, and progesterone **(D)** levels in the muskrats during the breeding season (BS) and non-breeding season (NBS). *Statistically significant values (*P < 0.05, **P < 0.01).

The distributions of hormones (FSH, LH, progesterone, and 17β-estradiol) are displayed in **Figure 8**. Compared to the samples from the BS, the levels of these hormones of the female muskrats were all remarkably lower than those from the NBS.

## Discussion

The present paper may be the initial research to take the female muskrat ovaries as the objects to examine the distribution patterns and expression of UPR signal genes and steroidogenic enzymes both from the BS and the NBS. These findings uncovered that the mRNA levels of GRP78, ATF4, ATF6, XBP1s, P450scc, P450arom, P450c17, and 3β-HSD were considerably higher in ovaries from the BS in comparison with those from the NBS, while, there were no considerable differences CHOP and CASP3 between the BS and the NBS at the mRNA level. In comparison with the female muskrats from the NBS, the concentrations of LH, FSH, 17β-estradiol, and progesterone were greater in female muskrats from the BS. Furthermore, RNA-seq data from the ovaries collected across the various breeding seasons showed clearly that DEGs mapped in a variety of pathways, including those associated with ERS and ovarian steroidogenesis signaling pathway with the aid of enrichment analysis. These results indicated that UPR signaling associated with the seasonal changes in ovarian steroidogenesis is activated in the breeding season and the delicate balance in redox regulation is important for seasonal reproduction in the muskrats.

When compared to the samples from the NBS, the ovarian size of the female muskrats was larger, as well as the ovarian weight of the female muskrats was heavier than those from the NBS in this study. According to histological observations, the ovaries of female muskrats from the BS had primary, secondary, antral, and dominant follicles, while those of ovarian tissues that were from the NBS only contained preantral follicles. This revealed that preantral follicles in the ovaries of female muskrats during the NBS could not continue to develop into post-antral follicles or even mature into corpus luteum. These findings are consistent with other seasonally breeding animals. In wild female ground squirrels, when compared to the ovaries from the BS, the number of secondary follicles, antral follicles, post-antral follicles, and corpus luteum considerably decreased in the NBS (34). The form and function of the ovaries of buffaloes were affected by reproductive status and season (35). During the period of seasonal infertility, the porcine oocytes were unable to fulfill their full developmental potential (36). The present research further provided strong evidence for the view that differences in follicular development existed between the BS and NBS in the ovaries of seasonally breeding mammals.

The UPR signaling, which protects against ER stress, is essential for animal development, female reproduction, the release of steroid hormones, and cellular homeostasis (37, 38). In this research, we observed the immunoreactivity of GRP78 localized in granulosa cells of mature follicles in ovaries from the BS, but not in granulosa cells of secondary follicles from the NBS, which is in accord with the study in the ovarian granulosa cells of the mouse (39). ER stress was activated in granulosa cells of late follicular development (large secondary, antral, and preovulatory) but not in granulosa cells of primary and small secondary follicles in mice (39). In addition, it has been demonstrated in numerous studies that persistent ER homeostasis disturbances during follicular growth and development cause the ERS response, which in turn triggers the UPR pathway, which in turn activates the apoptotic cascade. Yang et al. (40) demonstrated that ERS regulates apoptosis of mouse ovarian granulosa cells by up-regulating the mRNA level of CHOP. Similar results in goat ovaries also demonstrated that the ERS-mediated apoptotic pathway is indeed involved in the apoptosis of granulosa cells (41). In the condition of polycystic ovary syndrome (PCOS), ERS can also trigger ovarian granulosa cell apoptosis (42). In this study, the mRNA levels of GRP78 in the ovaries from the BS were strikingly higher than those from the NBS, indicating higher levels of ERS in the breeding season, accompanied by the increased level of antioxidant enzymes (SOD2, CAT, and GPX1). The transcription levels of UPR signaling genes (ATF4, ATF6, and XBP1s) were also considerably higher in the ovaries from the NS in contrast to those in the NBS, suggesting that UPR signaling was activated by ERS in the muskrat ovaries. However, the transcription levels of CHOP and CASP3 in muskrat ovaries were not obviously different between the BS and the NBS, indicating that the apoptosis level was not greater in the BS than in the NBS. A similar study was confirmed in an *in vitro* model of bovine oocytes, where it was shown that oxidative stress and UPR responses were exhibited in surviving blastocysts (43). It was discovered that the female reproductive system was affected by the modulation of UPR signals and ERS by means of the preservation of homeostasis in the cells and the beginning of apoptosis (44). The activation of the UPR by the modest levels of ERS in granulosa cells and/or cumulus cells may aid in the maturation of human oocytes (45). These results suggest that during normal follicular maturation and ovulation, ERS and the short-term UPR signaling as an adaptive response are expressed at higher levels during the breeding season, are beneficial and necessary in the muskrat ovaries, which may contribute to the production of large amounts of steroid hormones during the breeding season.

For the past few years, a growing number of researches have focused on the ERS in inextricable association with steroid hormones (46, 47). ERS not only regulates follicular development and atresia but is also critical for steroid hormone synthesis (47). As a functional tissue secreting hormones, the ovary is rich in the endoplasmic reticulum in follicular cells and luteal cells to maintain cholesterol synthesis, and normal endoplasmic reticulum function provides sufficient substrates for the synthesis of estrogen and progesterone (20, 48). Therefore, ERS-mediated UPR signaling may have a noteworthy effect on managing estrogen and progesterone production. In this study, in comparison with the samples from the BS, the levels of 17β-estrogen and progesterone in the serum of female muskrats from the NBS were considerably lowered, which were positively correlated with a notable decrease in LH and FSH concentrations when muskrat is in the NBS. In both cows (49) and mice (38), all three UPR signaling pathways were found to be activated in the luteal phase of the estrous cycle. The UPR activation-induced GRP78, ATF4, p50ATF6, and sXBP1 at the mRNA level were mostly maintained in functional and early regression stages of the mice corpus luteal (38). In the cows, the GRP78, ATF6, and XBP1 act as ER chaperones for initiating corpus luteal development and maintaining the corpus luteum, accompanied by elevated steroidogenic enzymes (49). Moreover, RNA-seq data from muskrat ovaries also confirmed the seasonal changes in ERS and steroid production, which revealed 2580 DEGs between the ovarian tissues from muskrats in the BS and NBS. In particular, there were 38 genes identified related to ER. Through further analysis of the intersection genes, we found that the ER-related pathways were engaged in the seasonal reproduction of female muskrats, and MAPK, PI3K-Akt, and ovarian steroidogenesis signaling pathways were obviously enriched by means of the KEGG analysis. Of which, the PI3K/Akt and MAPK are the most represented in the number of genes concerned. This suggests that Insulin or IGF-1 might be involved. In this study, the rise in ERS and UPR signaling was accompanied by the increased expressions of steroidogenic enzymes. Therefore, inferred from these results is that ERS may have an influence on controlling seasonal variations in steroid hormone synthesis and secretion in the ovaries of muskrats.

Some studies have also shown that the dynamic changes of ERS-related molecules seem to have some connection with steroidogenic enzymes, suggesting that UPR may affect the expression of steroid synthase (49, 50). The ATF6 pathway, which is activated by hCG, is crucial for the production of steroidogenic enzymes, particularly 3β-HSD, in Leydig cells (50). It was demonstrated that rat granulosa cells had a very high level of GRP78 expression and that ovulation stimulation, such as LH or hCG, caused GRP78 expression in these cells along with an up-regulation of LHR (51). It was found that the knockdown of XBP1 by RNAi in mouse granulosa cells reduced the expression level of P450arom and resulted in decreased estradiol secretion (52). The synthesis of progesterone and estradiol in mouse granulosa cells was clearly boosted by ATF6 knockdown, in keeping with the up-regulation of P450scc, StAR, and P450arom mRNA levels (53). Based on these evidences and our results, we suggest that the ovaries of muskrats are regulated by gonadotropins acting on the pituitary gland, and secrete large amounts of proteins and sex hormones, which are positively correlated with the high level of ERS-induced UPR signaling during the breeding season.

In conclusion, significant changes in steroid hormones and ERS occurred in the ovaries of female muskrats during seasonal reproduction. During the breeding season, with the continuous growth in follicular size and the massive proliferation of granulosa cells in the follicle, the high secretory demand of the endoplasmic reticulum (e.g., steroid hormones and steroidogenic enzymes) becomes one of the reasons for the increase in ERS. And ERS-induced UPR signaling was remarkably elevated in the muskrat ovaries during the breeding season to maintain cellular homeostasis. The results collected provide fresh avenues for investigating the role of ERS in female reproduction function and may reveal how it regulates reproduction in different seasons (for seasonally breeding mammals) and responds to varied influences (reproductive aging, stress, etc.). We intend to investigate the precise mechanism of the probable pathway in ERS impacts *in vitro* on the function of the ovary in muskrats in the future.

## Data availability statement

The original contributions presented in the study are included in the article/**Supplementary Material**. Further inquiries can be directed to the corresponding author.

## Ethics statement

The animal study was reviewed and approved by the Ethics Committee of Experimental Animals at Beijing Forestry University.

## Author contributions

WL and QW designed the study. WL and JW performed the experiments. WL, QG, and WX collected, analyzed, and interpreted data. WL drafted the manuscript. YH, HZ, and ZY supervised the project. QW, YH, HZ, and ZY reviewed the manuscript. All authors contributed to the article and approved the submitted version.
