# Supplementary material for: Seasonal changes in endoplasmic reticulum stress and ovarian steroidogenesis in the muskrats (Ondatra zibethicus)
Source: Front Endocrinol (Lausanne). 2023 Feb 7;14:1123699. doi: 10.3389/fendo.2023.1123699 (PMC9941330; doi:10.3389/fendo.2023.1123699)
Supplement: Supplementary file 1 [file Image_1.pdf]

Supplementary Material

1.1 Supplementary Figures

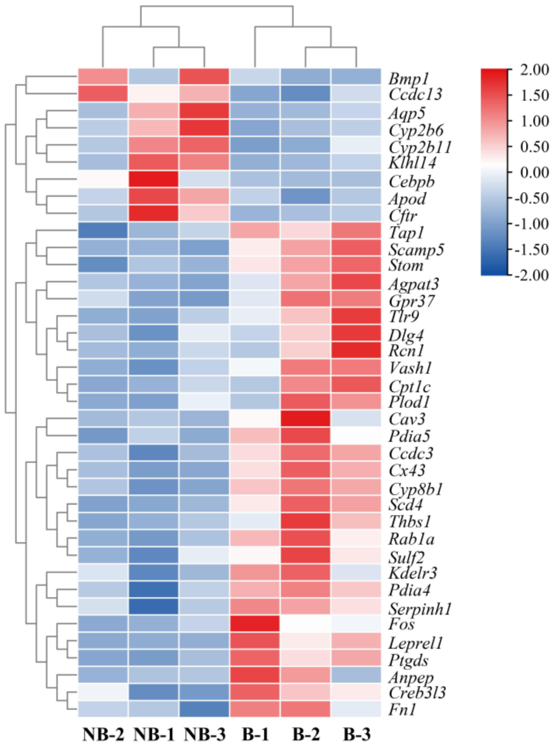

Supplementary Figure 1. Hierarchical clustering analysis of DEGs related to endoplasmic reticulum. B, breeding season; NB, non-breeding season.
